# Analysis of peripheral B cells and autoantibodies against the anti-nicotinic acetylcholine receptor derived from patients with myasthenia gravis using single-cell manipulation tools

**DOI:** 10.1371/journal.pone.0185976

**Published:** 2017-10-17

**Authors:** Tomohiro Makino, Ryuichi Nakamura, Maki Terakawa, Satoshi Muneoka, Kazuhiro Nagahira, Yuriko Nagane, Jyoji Yamate, Masakatsu Motomura, Kimiaki Utsugisawa

**Affiliations:** 1 Drug Discovery Technology Function, Asubio Pharma Co., LTD, Kobe, Japan; 2 Regenerative Medicine Field, Asubio Pharma Co., LTD, Kobe, Japan; 4 Immunology & Inflammatory Disease Field, Asubio Pharma Co., LTD, Kobe, Japan; 3 Laboratory of Veterinary Pathology, Osaka Prefecture University, Osaka, Japan; 5 Department of Neurology, Hanamaki General Hospital, Iwate, Japan; 6 Medical Engineering Course, Department of Engineering, the Faculty of Engineering, Nagasaki Institute of Applied Science, Nagasaki, Japan; Istanbul University, TURKEY

## Abstract

The majority of patients with myasthenia gravis (MG), an organ-specific autoimmune disease, harbor autoantibodies that attack the nicotinic acetylcholine receptor (nAChR-Abs) at the neuromuscular junction of skeletal muscles, resulting in muscle weakness. Single cell manipulation technologies coupled with genetic engineering are very powerful tools to examine T cell and B cell repertoires and the dynamics of adaptive immunity. These tools have been utilized to develop mAbs in parallel with hybridomas, phage display technologies and B-cell immortalization. By applying a single cell technology and novel high-throughput cell-based binding assays, we identified peripheral B cells that produce pathogenic nAChR-Abs in patients with MG. Although anti-nAChR antibodies produced by individual peripheral B cells generally exhibited low binding affinity for the α-subunit of the nAChR and great sequence diversity, a small fraction of these antibodies bound with high affinity to native-structured nAChRs on cell surfaces. B12L, one such Ab isolated here, competed with a rat Ab (mAb35) for binding to the human nAChR and thus considered to recognize the main immunogenic region (MIR). By evaluating the Ab in *in vitro* cell-based assays and an *in vivo* rat passive transfer model, B12L was found to act as a pathogenic Ab in rodents and presumably in humans.These findings suggest that B cells in peripheral blood may impact MG pathogenicity. Our methodology can be applied not only to validate pathogenic Abs as molecular target of MG treatment, but also to discover and analyze Ab production systems in other human diseases.

## Introduction

Myasthenia gravis (MG) is an autoimmune disease characterized by fluctuating muscle weakness and abnormal fatigue in those affected [1–3]. It is mediated by Abs that target antigens located at neuromuscular junctions (NMJs) of skeletal muscle [4–6]. Around 85% of patients with MG possess autoantibodies against the adult form of the muscle nicotinic acetylcholine receptor (anti-nAChR Abs) [4,5]. By analyzing mAbs isolated from antigen-immunized rats via hybridoma technology, anti-nAChR Abs and their pathogenic mechanism in rodents have been extensively characterized [5,7]. In addition, a passive transfer model of experimental autoimmune MG (EAMG) mediated by monoclonal and polyclonal Abs has also contributed fundamentally to our understanding of the pathogenic mechanism underlying MG [5,7,8]. Binding of these Abs to the receptors triggers a decrease in receptor density by inducing complement-dependent cytotoxicity, downmodulating the receptors on the cell surface, or even antagonizing receptor function [6,7].

The receptor nAChR, in muscles consists of a heteropentamer (two α-subunits and one each of β-, δ-subunit, and γ-subunit [embryonic type] or ε-subunit [adult type]) organized around a central pore in the membrane [9,10]. On average, more than 50% of the binding activity of Abs against nAChR in the sera of patients with MG was blocked by each mAb raised in rats (mAb35) or humans (mAb637). In addition, the epitopes of both Abs are located at the top of the nAChR α-subunit, called the main immunogenic region (MIR) [11,12]. Rat mAb35 is known as one such MIR Ab [13,14]. Several articles have described the isolation of anti-nAChR Abs from humanized mice and patients with MG by using phage display techniques or the Epstein-Barr virus [11,12,15–18]. However, the extent of the human repertoire of anti-nAChR Abs remains unknown because of limitations in the technologies available to date.

Single cell manipulation technologies have improved dramatically in recent years and have been applied in many fields such as analytical chemistry, chemical engineering, and biomedical science [19–21]. They have shed a light on the acquired immune system, including B and T cell repertoires and the dynamics of their responses to infections and in autoimmune diseases [22–24], which could not have been achieved using conventional technologies such as phage display and hybridomas. These technologies have also been utilized to discover Abs for diagnostic and therapeutic purposes [25,26].

In this study, by employing a single cell manipulation tool and direct preparation of recombinant Abs [27,28] coupled with novel high-throughput cell-based binding assays, we successfully analyzed the anti-AChR Ab repertoire derived from individual peripheral B cells of patients with MG and isolated a pathogenic Ab which can be a molecular target for MG therapy in human. We found that most of the Abs showed low binding affinities for nAChRs and diverse amino acid sequences in complementarity determining regions (CDRs). In addition, by sorting memory B cells by fluorescent antigen (a recombinant extracellular domain [ECD] of the α-subunit of human nAChR), we isolated some mAbs that specifically recognized the nAChR in a conformation-dependent manner. One such mAb, B12L, which competed with mAb35, showed the highest affinity for nAChRs and induced a myasthenic phenotype in a passive transfer rat model.

## Materials and methods

### Cell lines, yeast strain, *Escherichia coli* strain

Expi 293, purchased from Thermo Fisher Scientific, was used to express recombinant Abs. The human rhabdomyosarcoma cell line TE671, which expresses human nAChR on its surface [29,30], and DB40, transfected with δ-subunit genes to CN21 (cells transfected with the ε-subunit of TE671) for the stable expression of both fetal and adult AChRs [31,32], were purchased from the American Type Culture Collection (ATCC) and ISIS Innovation Ltd., Oxford, respectively. These cell lines were used for *in vitro* assays, including the binding, AlphaLISA, downmodulation, antagonist/agonist, and competition assays.

*Pichia pastoris* (GS115), purchased from Thermo Fisher Scientific, was used to express the ECD of the α-subunit of human nAChR for ELISA and cell sorting.

*E*. *coli strain* TOP10 (Thermo Fisher Scientific) was transformed with mAb35xich1 and B12L expression vectors.

### Antibodies and reagents

Anti-human CD19-FITC, CD-19- Brilliant Violet 421, anti-human CD38-Brilliant Violet 421, and anti-human CD27-APC (Allophycocyanin) were purchased from Miltenyi Biotec. Anti-human IgG-PE and IgG-APC were acquired from BioLegend. HRP-conjugated anti-human IgG (heavy and light chains) and isotype-matched human IgG were purchased from Jackson ImmunoResearch Laboratories. Anti-rat C3 Ab (clone 12E2) and anti-human synaptic vesicle protein 2A (SV2A) Ab were purchased from Novus Biologicals. Alexa Fluor 488 anti-mouse Ab and Alexa Fluor 488 anti-rabbit Ab were obtained from Life Technologies.

EAMG sera from rats immunized with AChR from *Torpedo californica*, extract of the electric organ of *Torpedo californica*, and the rat sciatic nerve were kindly gifted by the University of Nagasaki, Japan.

### Plasma and PBMCs from patients with MG and healthy controls

All samples were obtained after receiving written informed consent from patients. Plasma titers of Ab against nAChR were measured using a clinical diagnostic RIA kit (Cosmic Corporation, Japan). All experiments were conducted in accordance with the Declaration of Helsinki. The study protocols (Permit Number: # HG-12-001, # HT-15-015, and #E-12-006) were approved by the ethics committees of Hanamaki General Hospital, Iwate, Japan and Asubio Pharma Co., Ltd, Kobe, Japan. Plasma and PBMCs derived from patients with MG were obtained from the Hanamaki General Hospital, and PBMCs derived from a healthy volunteer were purchased from Veritas Corporation, Tokyo in a cryopreserved condition. The recovery rate of plasmablasts from cryopreserved PBMCs might be lower than that of plasmablasts from fresh PBMCs.

### Flow cytometry

A FACS Aria™ III (Becton Dickinson) was used to isolate peripheral B cells from PBMCs. MACSQuant Analyzers (Miltenyi Biotec) was used for *in vitro* assays including binding, downmodulation of nAChR, and competitive activities.

### PBMCs isolation, cell staining and single cell isolation

PBMCs were isolated by centrifuging 20 mL of fresh blood overlaid with the same volume of 100% Ficoll (GE Healthcare) for 40 min at 400 × *g*. Memory B cell and the plasmablast fraction were separated using MACS isolation kit (Miltenyi Biotec), according to the manufacturer’s instructions. Approximately 5 × 10^6^ cells were washed with PBS and resuspended in 500 μL of FACS buffer (0.5% BSA and 0.05% azide in PBS). Cells were then stained with anti-human CD19-Brilliant Violet 421, anti-human IgG-APC and the recombinant ECD of the α-subunit of nAChR conjugated with PE to detect memory B cells [33–35]; and anti-human CD27-APC, CD19-FITC, and CD38-Brilliant Violet 421 for plasmablasts [36,37]. After 30 min of incubation on ice, cells were washed twice with FACS buffer and resuspended in 500 μL of the same buffer containing diluted 7-Amino-Actinomycin D (7-AAD) (BD Biosciences) prior to cell sorting.

Single CD19 ^++^ and IgG^++^ memory B cells with or without antigen^++^ (recombinant ECD of the α-subunit of human nAChR), as well as single CD27^++^, CD19^++^, and CD38^++^ plasmablasts, were sorted into each well of U-bottom 96-well plates containing 10 μL of cell lysis solution with 10 μg oligo-(dT)_25_ magnetic beads (Thermo Fisher Scientific) after excluding debris and dead cells based on scatter signals, 7-AAD fluorescence and doublet cells signals.

### Single cell manipulation and preparation of recombinant Abs

Human variable region of the heavy chain (VH) and variable region of the light chain (VL) genes were amplified from each sorted cell as previously described [27]. An automatic magnetic empowered transmission instrument was engaged for the preparation of the 3′-end homopolymer-tailed cDNA from single B cells [27,28]. The VH and VL genes were then amplified by 5′ RACE nested PCR using the 3′-end homopolymer-tailed cDNA fragments as templates. Subsequently, the PCR-amplified V gene fragments were joined to their respective DNA cassettes to build linear immunoglobulin heavy chain (IgH) and light chain (IgL) genes by target selective-joint PCR (TS-jPCR) [27,28]. All PCR products were analyzed at each step on Qiaxcel capillary agarose gel (Qiagen).

The pairs of IgH and IgL gene cassettes prepared above were co-transfected using ExpiFectamine™ 293 transfection reagent (Thermo Fisher Scientific) into 2.8 ×10^6^ cells/700 μL of Expi293 cells (Thermo Fisher Scientific), then the cells were grown in 96-well deep culture blocks (Corning) with appropriate agitation. After five days of incubation at 37°C and 8% CO_2_, the supernatants containing secreted recombinant Abs were then filtered through membranes with 0.22-μm pores and analyzed in each *in vitro* cell-based assay. The concentration of Abs in the supernatant was determined and compared with that of mock plasmids, using either a Dip and Read Protein G (ProG) Biosensors (ForteBio) or Human IgG ELISA Quantitation Kit (Bethyl Laboratories, Inc.).

For the construction of human chimeric rat anti- nAChR Ab (mAb35xich1), the VH/VL DNA sequence of mAb35 was amplified directly from cDNA derived from a mAb35 hybridoma (clone ATCC TIB-175™) using conventional methods. After this, the fragments were cloned into pSF-CMV-HuIGG1 HC/HuKappa LC (Sigma-Aldrich) carrying human IgG1 and Igk framework with an NcoI/XbaI restriction site.

To prepare large amount of B12L Ab for *in vivo* experiments, VH/VL fragments carrying IgG1 and Igλ, which were obtained after TS-jPCR were recloned into pcDNA 3.4 via TOPO cloning (Thermo Fisher Scientific). The purified paired plasmids IgH and IgL were then co-transfected in 30 mL or 150 mL Expi293 cells according to the manufacturer’s instructions. After five days of incubation, culture supernatant was harvested and clarified by centrifugation. Subsequently, Abs were purified and concentrated by HiTrap Protein G HP 5mL (GE Healthcare) following the manufacturer’s instructions. Fab fragments of mAb35 and B12L Abs were obtained using a Pierce™ Fab Preparation Kit (Thermo Fisher Scientific) according to the manufacturer’s instructions. The purity of each Ab and its Fab was assessed by SDS-PAGE and Coomassie Brilliant Blue staining. Concentrations of Ab were determined using either a Dip and Read Protein G (ProG) Biosensors (ForteBio) or Human IgG ELISA Quantitation Kit (Bethyl Laboratories, Inc.).

### DNA sequence and Clustal Omega analysis

DNA sequences of VH/VL were analyzed by conventional Sanger sequencing. Sequence primers (5′-AGCCGGGAAGGTGTGCACGCCGCTG-3′, 5′-ACAACAGAGGCAGTTCCAGATTTCAACTGC-3′, and 5′- AGTGTGGCCTTGTTGGCTTG -3′) were used for analyzing the heavy chain, kappa light chain, and lambda light chain, respectively. Amino acid sequences of CDRs in VH regions were determined by submitting DNA sequences to the Igblast website (http://www.ncbi.nlm.nih.gov/igblast/) and compared with each other sequences from the Clustal Omega website (http://www.clustal.org/omega/) using the default settings. As for the sequences of the heavy and light chains of mAb35xich1, we confirmed that the sequences obtained were exactly the same as those previously reported [38].

### Antigen preparation for the fluorescence probe and ELISA

The recombinant ECD of the α-subunit of human nAChR was prepared according to published methods [39,40] with minor modifications. cDNA encoding the ECD of the human α-subunit (1–211 aa) was obtained from NITE Biological Resource Center (NBRC) and cloned into pPICZα (Thermo Fisher Scientific) for expression in *P*. *pastoris* (GS115). Point mutation V8E and hexahistidine tags in the N-terminus of the ECD were introduced to stabilize the structure and purify the protein. For the fluorescence probe used in single cell sorting, purified protein was labeled with PE using an R-Phycoerythrin Labeling Kit -NH_2_ (Dojindo Laboratories).

### ELISA to detect the ECD of the α-subunit of human nAChR

Two hundred nanograms of purified ECD of the human α-subunit was immobilized on 96-well Maxisorp plates (Sigma-Aldrich) for 16 h at 4°C, followed by blocking with 200 μL of BlockAce (DS Pharma Biomedical Co., Ltd.) solution; 1% BSA was also immobilized as a reference. Culture supernatant or purified Abs (100μL each) was added at the appropriate dilution and allowed to bind for 1 h at 24°C followed by triple washing with TBS-T (Tris-buffered saline, 0.1% Tween 20). One hundred microliters of HRP-conjugated anti-human IgG (heavy and light chains) Abs (Jackson ImmunoResearch Laboratories) diluted 1:2000 in TBS-T were then added and allowed to bind for 1 h. After discarding the content of the wells, ELISA plates were visualized by adding 100 μL of 1-step Ultra TMB-ELISA (Thermo Fisher Scientific) and quenched with the addition of 100 μL/well of 1 N H_2_SO_4_. The OD_450_ was determined by plate reader and digitalized. Serially diluted mAb35xich1 and the isotype-matched IgG were used for positive and negative controls, respectively.

### Preparation of DB40/TE671 cell cultures for *in vitro* assays

Cell lines TE671 (ATCC) and DB40 (ISIS Innovation Ltd.) were cultured at 37°C and 5% CO_2_, with RPMI 1640 + 10% FBS or DMEM + 10% FBS+ 0.5 mg/ml of G418, respectively, in T75 flasks.

### Cell AlphaLISA

Cell AlphaLISA was performed as described in Bielefeld-Sevigny [41] with following modifications. Cells were detached with non-enzymatic dissociation solution (Sigma-Aldrich) and suspended in 1× AlphaLISA immunoassay buffer (25 mM HEPES, pH 7.4, 0.1% casein, 1 mg/mL Dextran500, 0.5% Triton X-100 and 0.05% Proclin-300). In each well of a 1/2 AreaPlate-96, 2× 10^4^ cells in 10 μL of buffer were mixed with 10 μL each of 0.1 μg/mL biotin-α-Bungarotoxin (biotin-α-Btx) (Thermo Fisher Scientific) and streptavidin-coated AlphaScreen Donor Beads (PerkinElmer). After incubation for 1 h at 24°C, 10 μL of culture supernatant and the same volume of anti-human IgG AlphaLISA Acceptor Beads (PerkinElmer) were added and incubated for another 1 h at 24°C. Data were obtained by measuring chemiluminescent emission at 615 nm using Envision (PerkinElmer). Serial diluted mAb35xich1 and isotype-matched IgG were used for positive and negative controls, respectively. All reagents and samples were reduced by half when AlphaPlate-384 was used for the assay.

### Flow cytometry (FCM) based binding assay and competition assay

Cells were detached by non-enzymatic dissociation solution (Sigma-Aldrich) from the culture flask and 5 × 10^4^ cells/well were incubated with 50 μL of diluted culture supernatant, purified Abs or MG plasma fractions in V-shaped 96-well plates for 30 min on ice. After washing with FACS buffer twice, 100 μL of PE-conjugated goat anti-human IgG (Fc) Abs (BioLegend) diluted 1:200 in FACS buffer was then added and allowed to bind for another 30 min on ice. After a washing step, cells were resuspended in 100 μL of FACS buffer containing diluted 7-AAD (BD Biosciences) before FCM scanning. The mean fluorescence signal of PE (570 nm) was collected after eliminating debris and dead cells based on scatter signals and 7-AAD fluorescence, and the signal intensities of DB40 and TE671 were compared. Serially diluted mAb35xich1 and isotype-matched IgG were used for positive and negative controls, respectively. The competitive inhibition assay was carried out similarly, except for the addition of a pre-incubation step: purified Fab was held for 30 min on ice before adding the MG plasma sample or Abs to be tested.

### FCM-based nAChR modulation assay

Downmodulation of nAChRs on the surface of DB40 cells was monitored based on a method described by Lozier BK et al. [31] with some modifications, particularly in detection. DB40 cells were detached with non-enzymatic dissociation solution (Sigma-Aldrich) from the culture flask and 5× 10^4^ cells/well were resuspended in 200 μL of DMEM with 10% FBS in V-shaped 96-well plates. Abs (mAb35xich1, mAb35-Fab, and B12L) or MG patients’ plasma were added at the appropriate concentrations. Cells were then incubated for 4 h at 37°C and 8% CO_2_. Fluorescein-labeled antagonists (α-Btx conjugated with Alexa Fluor 488 [α-Btx-Alexa Fluor 488, Thermo Fisher Scientific]) were used to monitor the amount of nAChR on the surface of cells. After incubation, cells were labeled with α-Btx-Alexa Fluor 488 (2 μg/mL final concentration) for 30 min on ice and washed with FACS buffer before FCM scanning. Data are presented as the mean fluorescence signal of Alexa Fluor 488 (530 nm) after eliminating debris and dead cells based on scatter signals and 7-AAD fluorescence. Serially diluted mAb35xich1 and isotype-matched IgG were used for positive and negative controls, respectively.

### Agonistic and antagonistic activity assays

Antibodies’ agonistic and antagonistic effects toward nAChRs were measured in accordance with the methods of Arias HR et al [42], and Fitch RW et al [43]. For the agonistic activity assay, DB40 cells (2 ×10^4^ cells/well) were grown overnight in 96-well black flat plates coated with Poly-d-Lysine (Corning). Serially diluted Abs were mixed and incubated for another 2 h, and then the Ca^++^ influx into cells was visualized using a FLIPR Calcium 6 Assay Kit (Molecular Devices). Signals of Ca^++^ influx were monitored with a FlexStation (Molecular Devices). Acetylcholine (Sigma-Aldrich) was used as a positive control. To evaluate the antagonistic activity of Abs against acetylcholine toward nAChR, serially diluted purified Abs and 1μM of atropine (Sigma-Aldrich), an inhibitor of the muscarinic type of AChR, were added 30 min prior to stimulation of acetylcholine. α-Btx (Thermo Fisher Scientific) was used as a positive control.

### Passive transfer of isolated Ab in rats

Animal experiments were approved by the Committee on Animal Experimentation (Permit Number: AEK-16-011R), and were carried out in accordance with the Guide for Animal Experimentation at Asubio Pharma Co., Ltd. All efforts were made to minimize animal suffering. Four-week-old female LEW/CrlCrlj rats were obtained from Charles River Laboratories Japan. These rats, weighing 102 ± 2 g, were divided into three groups and B12L Ab (1.5 mg/kg or 3.0 mg/kg) or saline (control) were administered intravenously to rats in each group (n = 3–4) after a week of acclimation.

Each animal was scored based on EAMG clinical scales (0, no weakness; 1, first signs of a weakened grasp after a few trials; 2, incomplete paralysis of hind limbs; 3, exhibiting generalized paralysis and moribund; 4, death) [14,44] at 3 h, 6 h, 9 h, 24 h, 30 h and 48 h after administration. Body weights were also measured at 24 h and 48 h after administration of the Ab or saline. In the view of humane endpoint, animals were euthanized immediately upon observation of a clinical scale of 3, and recorded as score 4 from then on. Euthanasia was performed by isoflurane anesthesia. In accordance with this criterion, we euthanized three animals at 30 h after 3 mg/kg B12L administration, and no animals were found dead in this experiment.

For the immunohistochemistry experiment, each animal (n = 4) was euthanized at 48 h after administration, under isoflurane anesthesia, and the extensor digitorum longus (EDL) tissue was subjected to immunohistochemistry. For the analysis of complement deposition, the rats were divided into two groups, and B12L Ab (1.5 mg/kg) or saline (control) were administered intravenously to rats in each group (n = 3). Each animal at 8 h after administration was euthanized under isoflurane anesthesia, and the EDL tissue was subjected to immunohistochemistry/immunofluorescence.

### Immunohistochemistry/immunofluorescence

EDLs were fixed with 1% paraformaldehyde (PFA)/PBS for 10 min and muscles were dissociated into fibers. Small pieces of these fibers were washed with 0.1M glycine/PBS for 30 min and blocked with 2% BSA/PBS for 60 min. To analyze complement deposition, muscle fibers were incubated with Alexa Fluor 594-α-Btx (Thermo Fisher Scientific) diluted 1:1000 in 2% BSA/PBS and mouse Ab against rat C3 (clone 12E2, Novus Biologicals, 1 μg/mL final concentration) for 16 h at 4°C. After triple washing with PBS, muscle fibers were incubated with Alexa Fluor 488 goat anti-mouse Ab (Life Technologies, 4 μg/mL final concentration). Following washing, muscle fibers were mounted on slide glasses and monitored with an Axio Scan Z1 (Carl Zeiss) or Fluorescence Microscope BZ-9000 (Keyence). To measure the amount of nAChRs in NMJs, muscle fibers were incubated with diluted Alexa Fluor 594-α-Btx (Thermo Fisher Scientific, 1 μg/mL final concentration) for 16 h at 4°C. Then, after soaking in methanol for 5 min, washing with PBS, and blocking for 60 min with 2% BSA/0.3% Triton-X100/PBS, muscle fibers were incubated with rabbit Ab against human SV2A (Novus Biologicals, 0.2 μg/mL final concentration). After washing with PBS three times, muscle fibers were incubated with Alexa Fluor 488 goat anti-rabbit Ab (Life Technologies, 4 μg/mL final concentration). The subsequent steps were the same as the procedures for analyzing complement deposition.

### Image analysis

ZEN imaging software (Carl Zeiss) was used for the image analysis. We selected four sites of region of interest (ROI), containing approximately 30 NMJs per animal, and measured the pixels of α-Btx and SV2A fluorescent signal. Then, the pixels of α-Btx positive signal colocalized with SV2A were divided by those of SV2A positive signal to calculate colocalization ratio of nAChRs to NMJs in these ROIs.

### Statistical analysis

Statistical analysis among the groups in certain figures were conducted using Dunnett’s multiple comparison test or Student’s *t*-test, with p < 0.01 being considered statistically significant. JMP®10.0.0, from SAS institute Inc., was used for the analyses.

## Results

### Isolation of memory B cells and plasmablasts from PBMCs by using FACS

**Fig 1:**
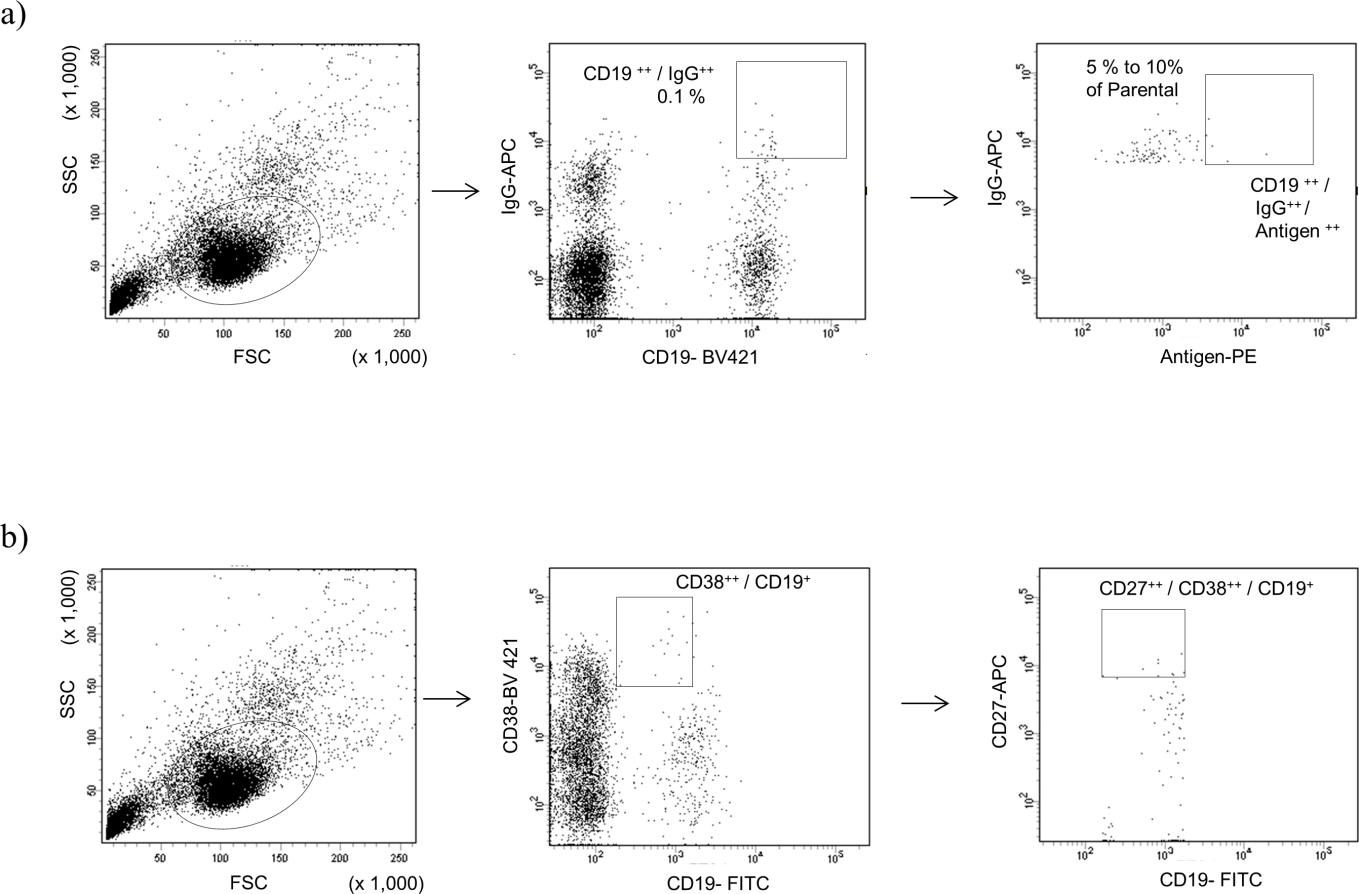
Scatter plot of flow cytometry analysis and gating strategy for single cell sorting of a) memory B cells with or without fluorescent-labeled antigen and b) plasmablasts.

Age, sex, serological results, clinical symptoms and MG Foundation of America (MGFA) clinical classification of donor patients with MG enrolled in this study are summarized in S1 Table. First, we isolated CD19^++^ IgG^++^ memory B cells and CD19^+^ CD27^++^ CD38^++^ plasmablasts from the PBMCs of five (MG1, 2, 3, 5, and 7) patients and one healthy control, and four (MG1, 2, 3, and 6) patients, respectively. Memory B cells were roughly 0.1% of PBMCs across the donors (Fig 1A). In contrast, the population of plasmabasts ranged from 0% to 0.2% across donors (0% for MG2, 0.02% for MG1, and 0.2% for MG3 and 6) (Fig 1B), which was consistent with previous observations [45].

Based on gating in FCM, we sorted several hundred single memory B cells from MG1, 2, 3, 5, and 7, as well as a healthy control; plasmablasts from MG3 and 6 were independently sorted into 96-well plates. Since the population of CD19^+^ CD27^++^ CD38^++^ plasmablasts was quite low or undetectable, we plated only 96 single cells from MG1 and could not harvest single cells from MG2 (S2 Table).

Methods to efficiently isolate B cells producing antigen-specific Abs include direct labeling and tetramer and secondary Ab methods [46,47]. In this study, we employed fluorescent-labeled antigen (recombinant ECD of the human nAChR α-subunit directly conjugated with PE) for multi-color cell sorting. Memory B cells with the highest fluorescent signals (5% to 10% of cells) were gated with CD19^++^ IgG^++^ cells (Fig 1A). The frequency of CD19^++^ IgG^++^ antigen^++^ cells was quite low, representing less than 0.01% of the parental population in all six samples.

### Amplification of IgH/IgL genes derived from individual memory B cells and plasmablasts in patients with MG

Several methods and technologies that allow manipulation of single cells have been reported for high-throughput isolation and cloning of mAbs [25,35,48]. In this study, we employed the magnetic-beads reaction through arrayed hanging droplets (MAGrahd) method, in which a magnetic power transmission system was used for single-cell-based cDNA synthesis and TS-jPCR was used for cloning, followed by seamless expression of IgH/IgL genes [27,28]. This method is suitable for analysis of genes from single cells (100 to 1,000 clones per donor) in a relatively inexpensive manner.

Human V_H_, V_Lκ_ and/or V_Lλ_ genes were successfully obtained and amplified by 5′ cDNA ends PCR (5′ RACE PCR) from single cells sorted in the gate as shown in Fig 1. The numbers and percentage of IgH/IgL genes, which were successfully amplified from isolated memory B cells, plasmablasts, and antigen^++^ memory B cells are summarized in S2 Table. We selected dozens of clones randomly and checked the sequences of amplified gene fragments, and confirming that each of them possessed unique human V_H_ and V_L_ sequences.

Fifty to ninety percent of cognate pairs of IgH/IgL genes were amplified from individual memory B cells, with or without antigen sorting (S2 Table). IgH/IgL genes from plasmablasts, on the other hand, were amplified at a lower rate than memory B cells, i.e., 27% to 60% (S2 Table). Around 10% of clones could not be amplified from any sample, presumably because of the inaccuracy of FCM sorting or cDNA synthesis and PCR errors. Immunoglobulin kappa light chains were reported to be predominant in normal human serum [49]; however, this was not observed in our analysis of single cells. We also found that two isotypes of the light chain fragment (VL_κ_ and VL_λ_) were simultaneously amplified in a single cell (approximately 10% of the population) in all samples, which might be due to the inaccuracy of FCM sorting or cloning errors, as reported previously [27].

Following the TS-jPCR reaction, cognate pairs of IgH/IgL genes were subsequently transfected in Expi293 cells to produce/secrete recombinant mAbs into the culture medium. The culture supernatant was clarified and subjected to several different *in vitro* assays. The expression levels of clones differed dramatically, ranging from 1μg/mL to 100 μg/mL as determined by Protein G biosensor or ELISA.

### Evaluation of binding specificity, affinity, and sequence variation in isolated recombinant mAbs

To evaluate the specificity and affinity of isolated recombinant mAbs against nAChRs, we established an antigen ELISA and two cell-based binding assays. For the antigen ELISA, we prepared the recombinant ECD of the α-subunit of human nAChR based on previous reports [39,40]. The recombinant ECD produced in *P*. *pastoris* was reported to exhibit the native structure [39,40]. However, rat mAb35, which could only recognize the native conformation of α-subunit [50,51], did not bind firmly to the antigen that we prepared for our ELISA system, whereas mAb210 known to bind both the native and denatured forms of the α-subunit [50], bound the antigen in our ELISA. Based on these observations, we assumed that the antigen we prepared was not properly folded or was partially denatured. In addition, we have prepared recombinant ECD produced in mammalian expression system (CHO cells), and got comparative results with those produced in yeast expression system. Since the expression level of ECD in *P*. *pastoris* was much higher than that in CHO cells, we chose to use *P*. *pastoris* for preparation of antigen for ELISA.

For the cell-based binding assay, we established two different assay formats for high-throughput screening, namely, Cell AlphaLISA and the FCM-based binding assay. AlphaLISA is a homogeneous ELISA system originally developed by PerkinElmer, that utilizes luminescent oxygen-channeling chemistry for detection [41,52]. We applied this system to a cell-based assay and named it the Cell AlphaLISA system. When nAChRs labeled with α-Btx-conjugated acceptor beads come close (within 100 nm) to the Abs labeled with donor beads, an emission at 615 nm was detected through a series of chemical reactions triggered by the interaction of acceptor and donor beads. DB40, derived from TE671, expressed clustered and structured fetal and adult forms of the nAChR [32]. We validated this system using mAb35/mAb210 Abs and DB40/TE671 cell lines, and found them compatible with the FCM-based binding assay (S1 Fig).

**Fig 2:**
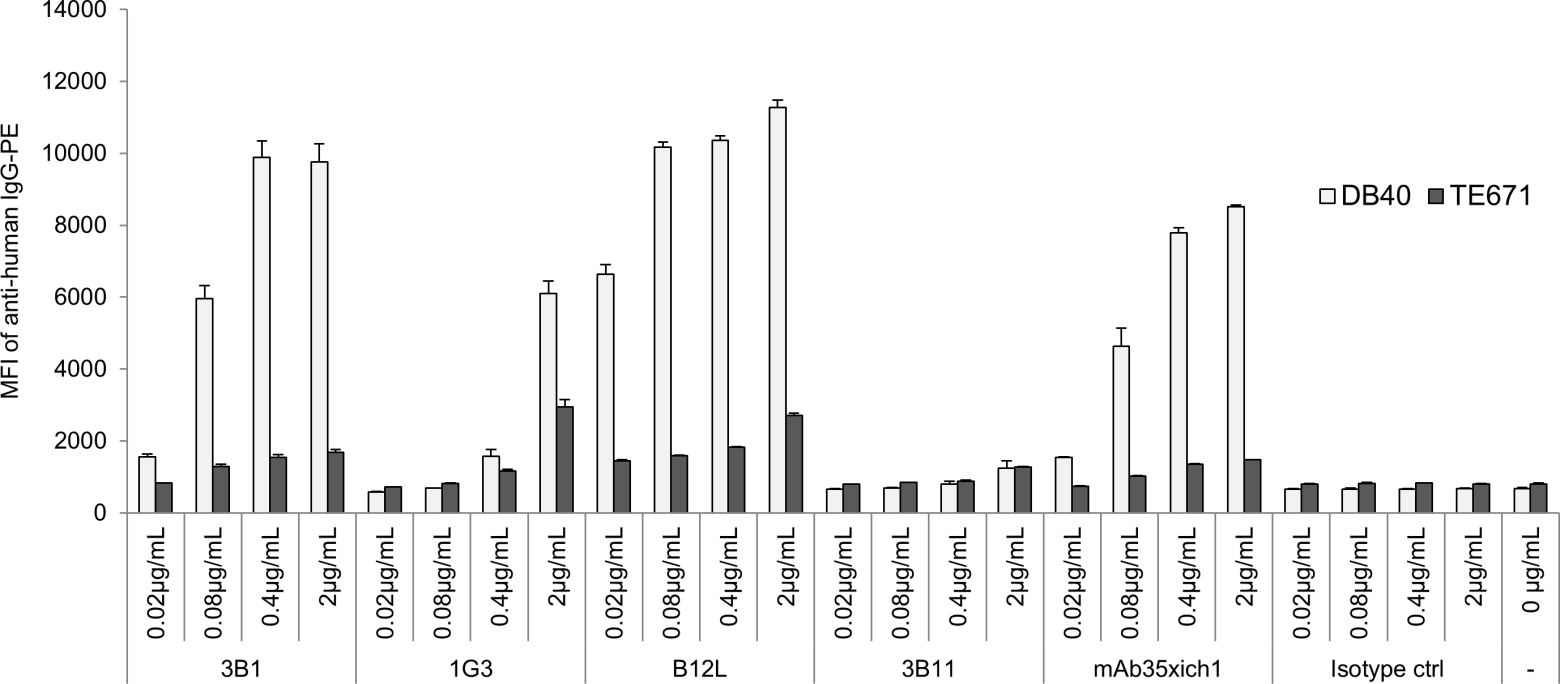
Binding of positive and negative clones to nAChR-expressing cells determined by flow cytometry based binding assay. Data shown are from experiments performed in triplicate and are shown as mean values with SD. y-axis: mean fluorescence intensity (MFI) of anti-human IgG-PE. 3B1, 1G3, B12L: positive clone; 3B11: negative clone; mAb35xich1: human chimeric form of rat mAb35 as positive control; isotype ctrl: human isotype-matched control Ab as a negative control. DB40: nAChR expressing cells; TE671: parent cell lines of DB40.

We evaluated all of the isolated clones by antigen ELISA and Cell AlphaLISA, and then selected the clones that showed a signal intensity greater than two-fold more than that of an irrelevant protein (BSA) or the parent cell line (TE671). Following this, we confirmed their binding to nAChR-expressing cells (DB40/ TE671) using an FCM-based binding assay. As shown in Tables 1 and 2, a certain amount (up to 5%) of Abs isolated from CD19 ^++^ IgG^++^ memory B cells and CD27^++^ CD19^++^ CD38^++^ plasmablasts from all MG donors bound to the recombinant ECD of the α-subunit in the antigen ELISA, whereas only one antibody out of 250 CD19 ^++^ IgG^++^ memory B cells from healthy control bound to recombinant ECD. Additionally, no single clone (from both MG donors and a healthy control) bound specifically to DB40 cells in the Cell AlphaLISA assay. We assumed that the majority of clones from MG donors could not bind to native receptors, but only bound to the unfolded form of ECD. An antibody from healthy controls that bound to the recombinant ECD was likely a non-specific binder, and such non-specific binders are rare, but could be present in samples from patients with MG to some degree. These results led us to perform the antigen–sorting of B cells (using the ECD of α-subunit) to acquire B cells producing anti-nAChR Abs more effectively. Before subjecting to the large scale sorting and analysis as shown in Table 3, we validated the antigen sorting system with small population of memory B cells from MG1 and MG7 donors. We found roughly ten times increase of in ELISA positive clones with antigen sorting relative to those without antigen sorting in MG1 (10/113 vs. 2/360; with vs. without antigen sorting) and MG7 (10/85 vs. 2/289; with vs. without antigen sorting). Among the Abs derived from CD19 ^++^ IgG^++^ antigen^++^ memory B cells in large scale sorting and analysis, a small fraction (less than 2%) of Abs that bound to DB40 specifically in a dose-dependent manner was detected in FCM-based binding assay (Table 3, Fig 2). As shown in Tables 1 and 3, eight cell-based-assay-positive clones out of 1015 were isolated with antigen sorting, whereas none of the clones was isolated without it, suggesting that we could enrich the fraction of B cells producing Abs against native nAChRs on cell surfaces using this antigen sorting method.

**Table 1:** Summary of positive clones derived from memory B cells.

Antigen ELISA and cell based binding assay		
Donor ID	Number of positive clones / Total clones (% of positives)	
	Antigen ELISA	Cell based binding assay
MG1	12 / 208 (5.8%)	0 / 208 (0%)
MG2	11 / 414 (2.7%)	0 / 414 (0%)
MG3	11 / 360 (3.1%)	0 / 360 (0%)
MG5	6 / 344 (1.7%)	0 / 344 (0%)
MG7	6 / 269 (2.2%)	0 / 269 (0%)
Healthy control	1 / 250 (0.4%)	0 / 250 (0%)
Total (MG)	46 / 1595 (2.9%)	0 / 1595 (0%)

ID indicates the donor enrolled in this study. Healthy control indicates non-MG PBMC purchased from the Veritas corporation. Number and % of positive clones of antigen (recombinant ECD of the α-subunit) ELISA and DB40 cell based binding assay are shown. Clones that show signal intensity greater than two-fold more than that of an irrelevant protein (BSA) or the parent cell line (TE671) are defined as positives.

**Table 2:** Summary of positive clones derived from plasmablasts.

Antigen ELISA and cell based binding assay		
Donor ID	Number of positive clones / Total clones (% of positives)	
	Antigen ELISA	Cell based binding assay
MG1	0 / 26 (0%)	0 / 26 (0%)
MG3	10 / 326 (3.1%)	0 / 417 (0%)
MG6	3 / 152 (2.0%)	0 / 360 (0%)
Total	13 / 504 (2.6%)	0 / 504 (0%)

ID indicates the donor enrolled in this study. Number and % of positive clones of antigen (recombinant ECD of the α-subunit) ELISA and DB40 cell based binding assay are shown. Clones that show signal intensity greater than two-fold more than that of an irrelevant protein (BSA) or the parent cell line (TE671) are defined as positives.

**Table 3:** Summary of positive clones derived from memory B cells with antigen sorting.

Cell based binding assay	
Donor ID	Number of positive clones / Total clones (% of positives)
MG1	2 / 198 (1.0%)
MG5	1 / 219 (0.5%)
MG7	1 / 178 (0.6%)
MG8	3 / 188 (1.6%)
MG10	1 / 192 (0.5%)
MG11	0 / 40 (0%)
Total	8 / 1015 (0.8%)

ID indicates the donor enrolled in this study. Number and % of positive clones of DB40 cell based binding assay are shown. Clones that show signal intensity greater than two-fold more than that of the parent cell line (TE671) are defined as positives.

DNA sequences from each positive clone were analyzed, and the CDRs in the VH region were compared using the Clustal Omega algorithm. We obtained 38/46, 12/13, and 8/8 DNA sequences from memory B cells, plasmablasts, and antigen-sorted memory B cells, respectively. These sequences were converted into amino acid sequences, and the regions of IgH CDRs were extracted using IgBlast. Sequences of CDR3 regions were then aligned and compared in Clustal Omega. As shown in S2 Fig, we did not find any conserved or similar sequences in the CDR3 regions of memory B cells or plasmablasts from donors. Although CDR3 of the VH domain is sufficient to define the specificity and affinity of most Abs [53], we compared CDR1 and 2 of IgH with the CDRs of IgL in the same way, and found no similarities (S3 Fig and S4 Fig).

### Characterization of human B12L, an anti-human nAChR Ab

**Fig 3:**
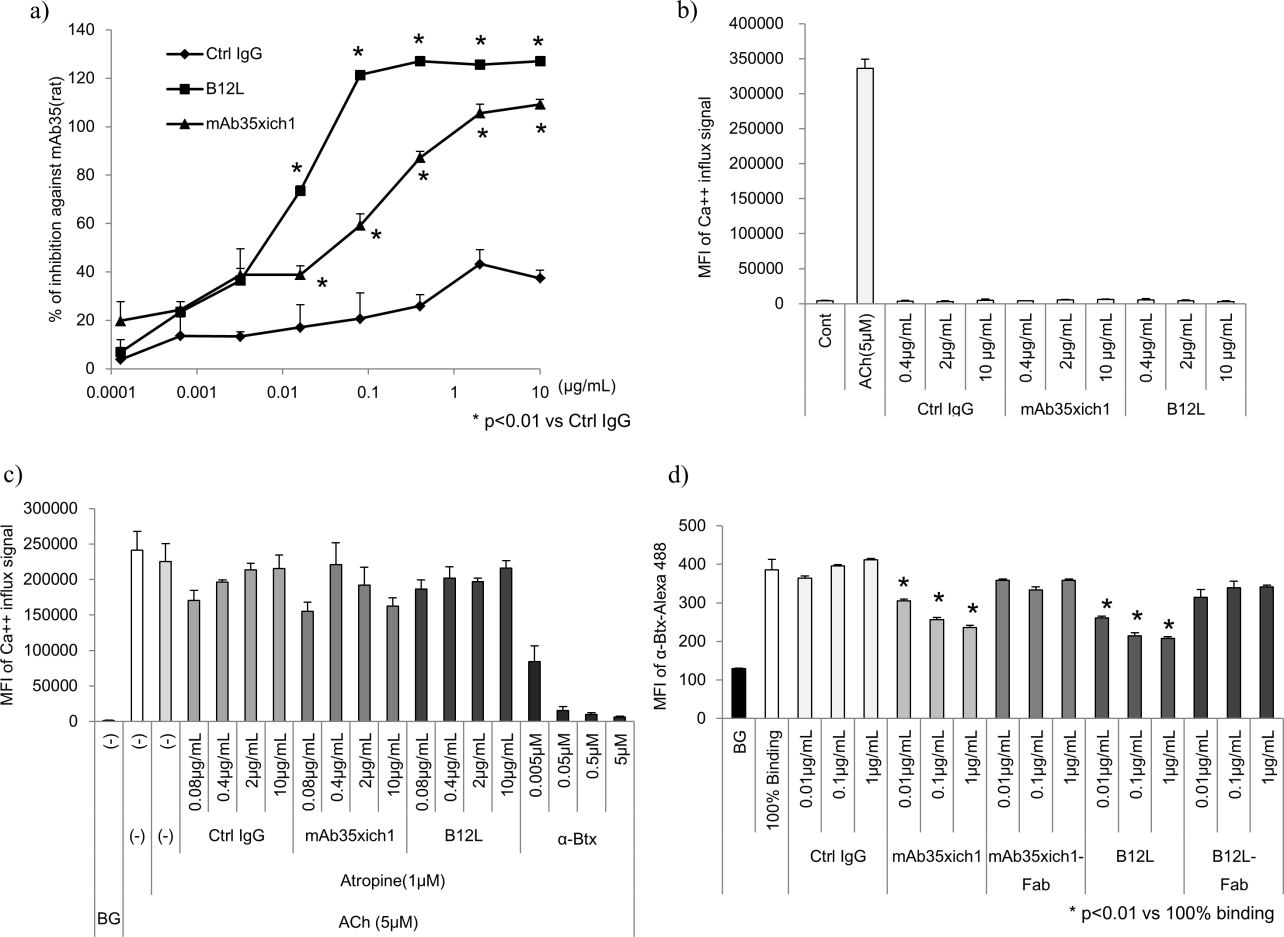
*In vitro* analysis of human B12L and rat mAb35. a) Competitive binding of rat mAb35 was monitored by flow cytometer-based binding assay. The indicated concentrations of Abs (mAb35xich1, B12L and Ctrl IgG) were added to DB40 cells prior to spiking with 10 μg/mL of rat mAb35. Data was obtained by monitoring the mean fluorescence intensity (MFI) of anti-rat IgG-PE signal. The percent inhibition was calculated with the following formula: (MFI of rat mAb35 signal without blocking Abs–MFI of rat mAb35 signal with blocking Abs) / (MFI of rat mAb35 signal without blocking Abs–background signal) x 100 (%). All data were obtained from experiments performed in triplicate and are presented as mean values with SD. Ctrl IgG: isotype-matched human IgG for negative ctrl IgG. b) Agonistic and c) antagonistic activities of B12L/mAb35 were monitored by Ca^++^ influx in DB40 cells. Acetylcholine (ACh) was used as a positive control agonist. Atropine was used as an inhibitor of muscarinic type AChR to reduce the background signal for antagonist activity. α-Btx was used as a positive control antagonist. d) Downmodulation of nAChRs induced by B12L, mAb35xich1, and their Fabs was monitored by fluorescence signal of the α-Btx-Alexa Fluor 488 probe. Binding at 100% represents the maximum fluorescence signal of α-Btx-Alexa Fluor 488 bound to nAChRs on the surface of DB40 cells. BG: background signal. Statistical analysis among groups in a) and d) were conducted using Student’s t-test, with p < 0.01 being considered statistically significant.

**Fig 4:**
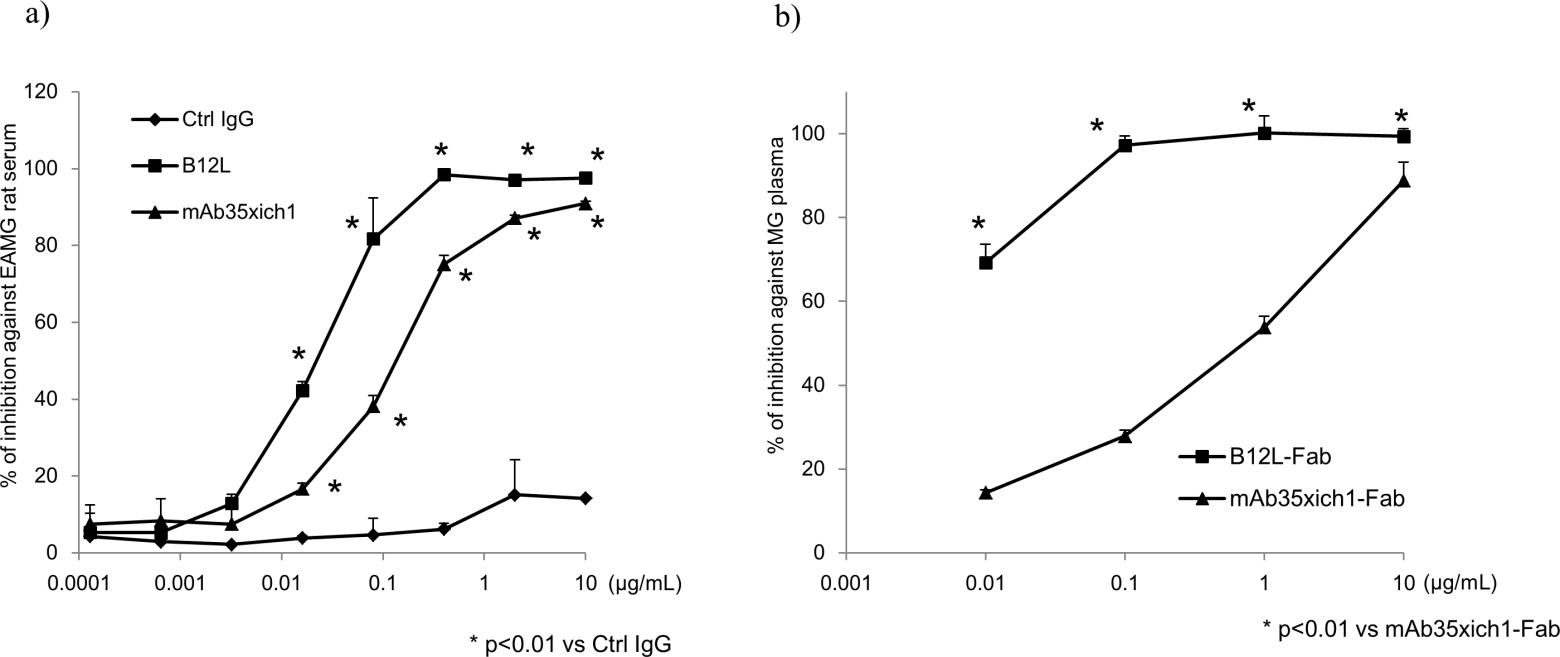
**In vitro competitive assay against a) rat EAMG serum and b) MG plasma.** a) The indicated concentrations of Abs (mAb35xich1, B12L and Ctrl IgG) were added to DB40 cells prior to spiking with rat EAMG serum. Data was taken by monitoring the mean fluorescence intensity (MFI) of anti-rat IgG-PE signal. b) The indicated concentrations of Fabs (mAb35xich1-Fab and B12L-Fab) were added to DB40 cells, prior to spiking with plasma from MG donor #1 (diluted 10 times). The percent inhibition was calculated as shown in Fig 3A. All data were obtained in triplicate after monitoring the MFI of anti-human IgG-PE signals through individual experiments, and are shown as mean values with SD. Ctrl IgG: isotype-matched human IgG for negative control IgG. Statistical analysis among groups in (a) and (b) were conducted using Dunnett’s multiple comparison test, with p < 0.01 being considered statistically significant.

To understand the nature of anti-nAChR Abs secreted from peripheral B cells in patients with MG, we examined one of the Abs, namely B12L, derived from MG donor #7 in more detail, because B12L emitted the highest signal of all Abs in the cell-based binding assay (Fig 2). The properties of B12L in comparison with rat mAb35 are summarized in Table 4. The epitope of B12L possibly overlapped or distinct but neighboring with that of mAb35 based on the results from the competitive inhibition assay (Fig 3A). In addition, there were some differences in their binding specificities to human, rat, and *Torpedo* nAChR (Table 4). Furthermore, B12L was able to downmodulate nAChRs and did not affect its function as an agonist/antagonist in the same fashion as mAb35 (Fig 3B, 3C and 3D). The maximum blocking rates of B12L against polyclonal Abs of EAMG rat serum and against four MG donors plasma, toward nAChR on cell surfaces were approximately 100%, 99%, 100%, 68%, and 100%, which were similar to the levels of mAb35 (98%, 91%, 94%, 62%, and 99%, respectively) (Fig 4, Table 4). However, B12L showed greater binding potency against nAChRs than mAb35, because the IC_50_ of B12L against EAMG serum and MG donor plasma was two orders of magnitude lower than that of mAb35 (Table 4). We have compared amino acid sequences of CDRs in VH region of these two Abs (S2D Fig), but could not find clear similarities between them. These results indicate that B12L generally exhibited similar binding as mAb35, but showed a preference for binding nAChRs over mAb35.

**Table 4:** Summary of characterization of B12L relative to mAb35.

	B12L	mAb35 (rat)
Subtype	human IgG1	rat IgG1
Origin	peripheral memory B	rat Hybridoma ^e)^
Epitope	MIR ^f)^	MIR ^g)^
Binding against Human nAChR ^a)^ Torpedo nAChR ^b)^ Rat nAChR ^c)^	+ + + - +	+ + + + + +
Competition against Rat EAMG serum Human MG plasma	100% (IC 50; 0.03 μg/mL) 99, 100, 68 and 100% ^h)^ (IC 50; 0.01 μg/mL)	98% (IC 50; 0.18 μg/mL) 91, 94, 62 and 99% ^h)^ (IC 50; 1 μg/mL)
Downmodulation ^d)^	Yes	Yes
Agonist/Antagonist	No / No	No / No

a) DB40 cell lines were used for human nAChR antigen, and relative binding affinity was determined by a flow cytometry (FCM)-based binding assay

b) extract from the electric organ of Torpedo californica were used for torpedo AChR antigen, and the relative binding affinity was determined by radio immunoprecipitation assay [54]

c) extract of rat sciatic nerve was used for rat nAChR antigen and the relative binding affinity was determined by radio immunoprecipitation assay [54]

d) downmodulation of full body Ab was determined by FCM-based assay, as shown in Fig 3F

e) originated from [12]

f) based on competitive binding by mAb35, as shown in Fig 3A

g) originated from [12]

h) maximum blocking potency against MG donors #1, 3, 5, and 7 are shown.

Plasma of MG1 was used after 10 times dilution with PBS, and those of MG3, 5, and 7 were used after 20 times dilution with PBS.

### Direct evidence of pathogenic features of B12L in the *in vivo* passive transfer rat model

**Fig 5:**
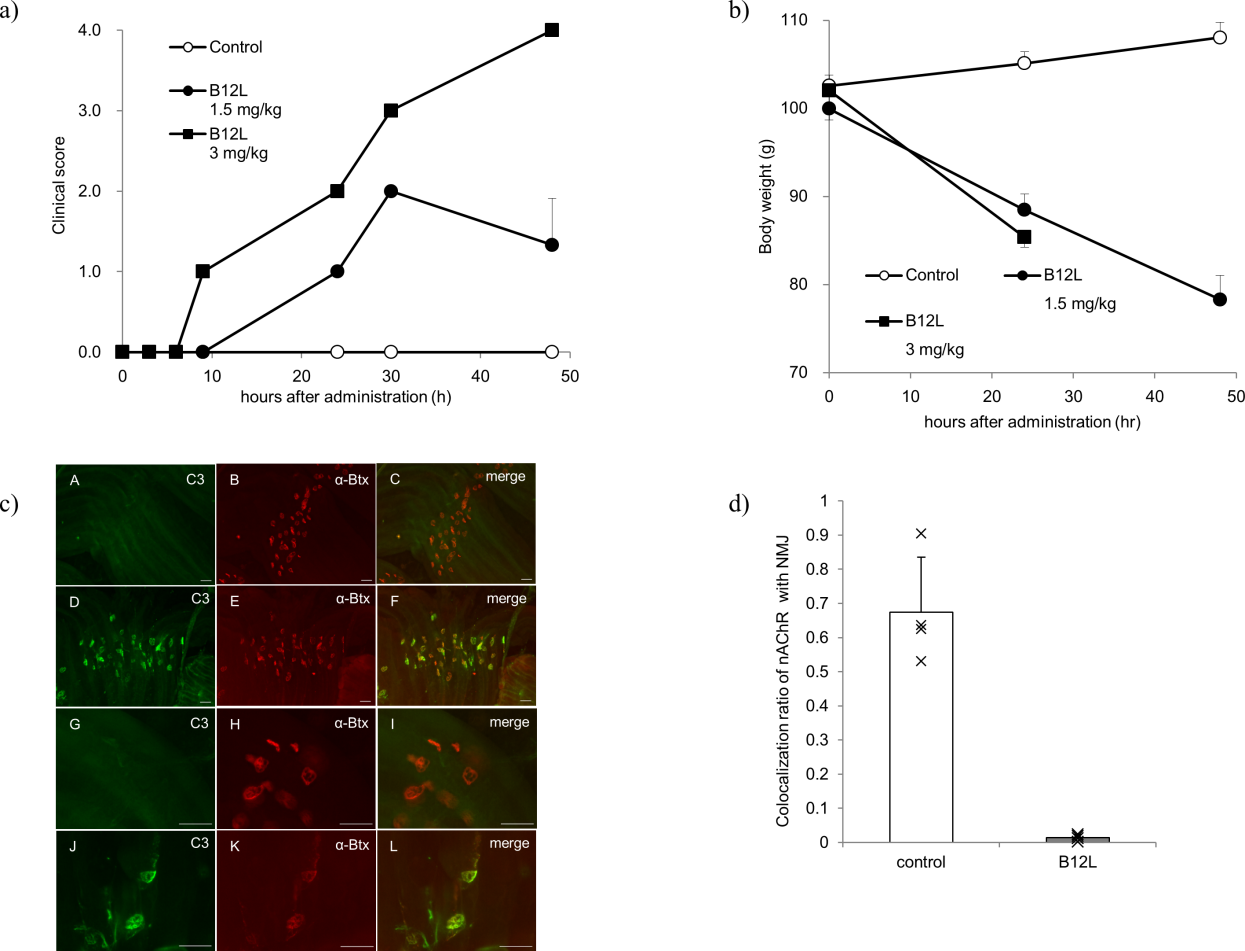
Passive transfer experiment for B12L. a) Clinical scores were recorded at 3 h, 6 h, 9 h, 24 h, 30 h, and 48 h after intravenous administration of B12L or saline (n = 3, each group, mean ± SD). Since the animals administered B12L at a dose of 3 mg/kg were moribund and euthanized 30 h after dosing, their clinical scores were recorded as ‘4’ at 48 h. b) Body weights were measured at 0 h, 24 h and 48 h after administration (n = 3, each group, mean±SD). We could not measure the body weights in the group administered 3 mg/kg of B12L at 48 h, because they were moribund and euthanized at 30 h after administration. c) Representative images of complement deposition in control rats (low magnification [A, B, and C], high magnification [G, H, and I]) and B12L-treated rats (low magnification [D, E, and F], high magnification [J, K, and L]) are shown. C3 (D and J, green) and α-Btx (E and K, red) signals were colocalized in neuromuscular junctions (NMJs) (F and L, merged) at 8 h after administration of B12L at a dose of 1.5 mg/kg. Scale bar = 50 μm. d) The amount of nAChRs in NMJs was calculated by imaging analysis. The graph shows the signal ratio of α-Btx (red) colocalized with SV2A (green) compared to SV2A signal alone in NMJs at 48 h after administration of saline or B12L at a dose of 1.5 mg/kg (n = 4, each group, mean±SD).

Lastly, we examined the MG pathogenicity of B12L in a passive transfer rat model. Administration of a single, intravenous dose of 1.5 and 3 mg/kg B12L to Lewis rats was sufficient to induce clear myasthenic phenotypes (muscular weakness and loss of weight) in a dose-dependent manner (Fig 5A and 5B). In the histological examination, at 8 h after injection of B12L, complement C3 was found to be colocalized with α-Btx-labeled nAChR at NMJs, whereas no deposition of complement C3 was observed in the saline control (Fig 5C). Then, at 48 h after injection, the nAChR signal was hardly detected at NMJs labeled with SV2A [55], suggesting that the number of nAChRs at NMJs was markedly decreased following complement deposition that was likely induced by B12L (Fig 5D). These results led us to conclude that B12L had pathogenic potential of MG both *in vitro* and *in vivo*.

## Discussion

Direct isolation of mAbs from individual cells that produce them in humans has long been hampered by the lack of suitable technologies such as the hybridoma technique in rodents [56,57]. Immortalization of B cells by Epstein-Barr virus infection showed some success, but was not widely applied because of the inefficiency of immortalization [57]. Surface display technologies such as phage, yeast, and ribosome displays have been very useful in the isolating of high-affinity Abs for therapeutic or research purposes [58]. However, these platforms were not the best approaches to examining B cell and Ab repertoires, because the natural pairing of IgH and IgL was often lost in the process of isolation. To study MG, there have been several attempts to isolate or examine anti-AChR Abs from the thymus or PBMCs using phage display [11,15,17]. Some anti-AChR Abs have been successfully isolated from humans, but this approach has failed to determine the abundance, preference, or natural pairing of IgH/IgL in these Abs. Recently, Vrolix and colleagues [18] successfully manipulated immortalized B cells derived from the thymus of patients with MG using Epstein-Barr virus infection. They isolated only one clone, out of 570 clones, which could specifically bind to fetal type nAChR. This method is very powerful, as B cells can be immortalized and expanded through *in vitro* cultures; however, it requires a higher biosafety level since it uses a human pathogen. Thus, in the present study, we developed a new methodology that employed a single cell manipulation system called MAGrahd [27,28] coupled with novel high-throughput cell-based binding assays to analyze several hundred single peripheral B cells and the Abs they produce, as well as their nAChR-antigen specificities and possible pathogenic roles in human MG. This sequential approach involved the following: isolating single B cells, amplifying the corresponding IgG, and evaluating its functions as a mAb. We also applied antigen sorting using directly labeled antigens (ECD of the α-subunit) to enhance the efficiency of isolating antigen-specific Ab-producing B cells [46,47]. This method is very robust and can be applied universally for the analysis of human Abs-production systems.

Using this methodology, we isolated various anti-AChR Abs, most of which recognized only denatured forms of the antigens. The populations of peripheral B cells that produced anti-nAChR Abs seemed to be low (less than 3% of the total memory B cells and plasmablasts) (Table 1). However, this frequency is significant, when compared to the frequency of non-specific binders obtained from the healthy control (0.4%). Thus, B cells producing non-specific binders are rare, but could be included to some degree, in PBMCs from patients with MG. But, when considering numerous kinds of other antigens against which memory B cells had probably been primed, it is unclear whether the frequency of anti-nAChR Ab-producing cell is really low. Furthermore, there seemed to be no clear correlation between the frequency of B cells or plasmablasts that produce such Abs and disease severity or serum anti-AChR Abs titers. In fact, only a very small fraction of memory B cells could produce anti-AChR Abs that could bind firmly to the native form of the receptor. These findings suggest that in the peripheral blood of MG patients, the majority of memory B cells or plasmablasts produce Abs directed against antigens other than nAChR, and that among a few nAChR-specific B cells, only a further limited fraction of these cells produce pathogenic Abs that can bind to structured nAChRs regardless of disease status. B cells producing anti-AChR Abs with low affinity to native-form receptors might be associated with epitope spreading or immature B cells. Further studies will be needed to address the physiologic or pathogenic roles of the Abs.

Rat mAb35, one of the best-characterized pathogenic Abs in MG originated from a rat EAMG model and recognizes MIR in native-structured α-subunit [10,50].

The MIR forms in part by 67–76 aa of conformationally correct α-subunits [51,59], although several other possible regions for the MIR have been suggested [15,60]. Human B12L, the mAb that showed the highest affinity for structured nAChR in the present study, probably shared an epitope with mAb35 and targeted the MIR in both humans and rats. Furthermore, B12L was clearly associated with MG pathogenicity in a passive transfer rat model, inducing complement deposition and a decrease in nAChR in NMJs. These lines of evidence suggest that a certain fraction of Abs produced by peripheral B cells, such as B12L play critical roles at least partly in the pathogenesis of human MG. There is a possibility that pathogenic Abs such as B12L will be target molecules in future MG therapeutics. The exact epitope of B12L should be identified by epitope mapping to clarify the differences or similarity among other anti-nAChR Abs, including mAb35. In addition, several other regi**o**ns may also function as the MIR; therefore, the functions of the other Abs that demonstrated some affinity to the structured nAChRs in the present study (e.g., 3B1 in Fig 2) should also be analyzed in the future.

The serum titers of nAChR Abs against MIR, determined by the mAb35 inhibition assay, tend to correlate with disease severity in patients with MG [54], suggesting the significance of MIR-specific nAChR Abs (e.g., human B12L) in the pathogenesis. However, the pathological significance of MIR-specific nAChR Ab-producing cells in the peripheral blood relative to those in lymphatic organs (the lymph node, spleen, or thymus) was not addressed in the present study. Quite recently, MGTX study revealed that thymectomy partly improved clinical outcomes in nonthymomatous MG patients [61], suggesting that, at least in some patients, the thymus probably is a resource of pathogenic nAChR Ab-producing B cells. However, Vrolix *et al*. analyzed B cells derived from thymus and found that the proportion of nAChR-specific antibodies was quite low (1/570) [18]. Thus, the precise correlation between pathogenic nAChR Abs in peripheral blood and disease status should be elucidated in the future study. However, the present findings support the lines of evidence that B cell depletion therapies, using anti-CD20 Abs, for example, can be effective in the treatment of MG [62] and that the serum titers of nAChR Abs are sometimes decreased but never eliminated solely by removal of thymic tissue [63,64].

According to our analysis of IgH CDRs, the amino acid sequences of isolated anti-AChR Abs were very diverse, showing little similarities in peripheral blood. In previous studies [36,65], autoantibodies in patients with pemphigus or neuromyelitis optica were shown to have CDRs with similar sequences, suggesting clonal expansion in peripheral blood. Although these two diseases are known to be driven by pathogenic autoantibodies, similar to anti-nAChR Abs in MG, there may be some differences in the antibody-production systems among these diseases [36].

## Conclusion

In the present study, we developed a single cell-based, comprehensive methodology that was applied to isolate and analyze peripheral B cells that produce pathogenic or non-pathogenic anti-nAChR Abs with native IgH/IgL. Using this methodology, we successfully isolated a clone of nAChR Abs against MIR, B12L from human MG samples and clearly demonstrated its pathogenicity in an EAMG model. In recent studies, blocking pathogenic antibodies against autoantigens using blocking antibodies [66,67] and depleting antigen-specific antibodies [68] were suggested as new concepts for MG therapy. Therefore, B12L could be a promising target molecule for MG therapy in the future. Our methodology can be applied universally to discover and analyze Ab production systems in other human diseases.

## Supporting information

S1 Fig Comparison of results from the flow cytometry (FCM)-based binding assay and Cell AlphaLISA. mAb35xich1 was used as a positive control. Data were obtained in triplicate from individual experiments and are shown as mean values with SD. y-axis; MFI of anti-human IgG-PE for the FCM-based binding assay, AlphaLISA signal for Cell AlphaLISA. DB40; nAChR expressing cells, TE671; parent cell lines of DB40
(TIF)

S2 Fig Alignment of IgH CDR3 amino acid sequences and analysis by Clustal Omega of ELISA-positive clones and flow cytometry-based binding assay-positive clones. Clone IDs and amino acid sequences shown are derived from memory B cells (a), plasmablasts (b) and antigen^++^ memory B cells (c). (d) Comparison of amino acid sequences of CDR1, 2 and 3 in IgH between mAb35 and B12L are shown.
(TIF)

S3 Fig Alignment of IgH CDR1 and 2 amino acid sequences and analysis by Clustal Omega of ELISA-positive clones derived from memory B cells (a), plasmablasts (b) and that of flow cytometry-based binding assay-positive clones derived from antigen++ memory B cells (c).
(TIF)

S4 Fig Alignment of IgL CDR1 and 2 amino acid sequences and analysis by Clustal Omega of ELISA-positive clones derived from memory B cells (a), plasmablasts (b) and that of flow cytometry-based binding assay-positive clones derived from antigen++ memory B cells (c).
(PDF)

S1 Table Age, sex, serological data, clinical symptoms and MGFA classification of MG donors enrolled in this study. (DOCX)

S2 Table Number and percentage of IgG genes amplified from a) peripheral memory B cells derived from MG donors, b) peripheral plasmablasts derived from MG donors, c) peripheral antigen^++^ memory B cells derived from MG donors.
(DOCX)
