# Supplementary figures and images for: Analysis of peripheral B cells and autoantibodies against the anti-nicotinic acetylcholine receptor derived from patients with myasthenia gravis using single-cell manipulation tools
Source: PLoS One. 2017 Oct 17;12(10):e0185976. doi: 10.1371/journal.pone.0185976 (PMC5645109; doi:10.1371/journal.pone.0185976)

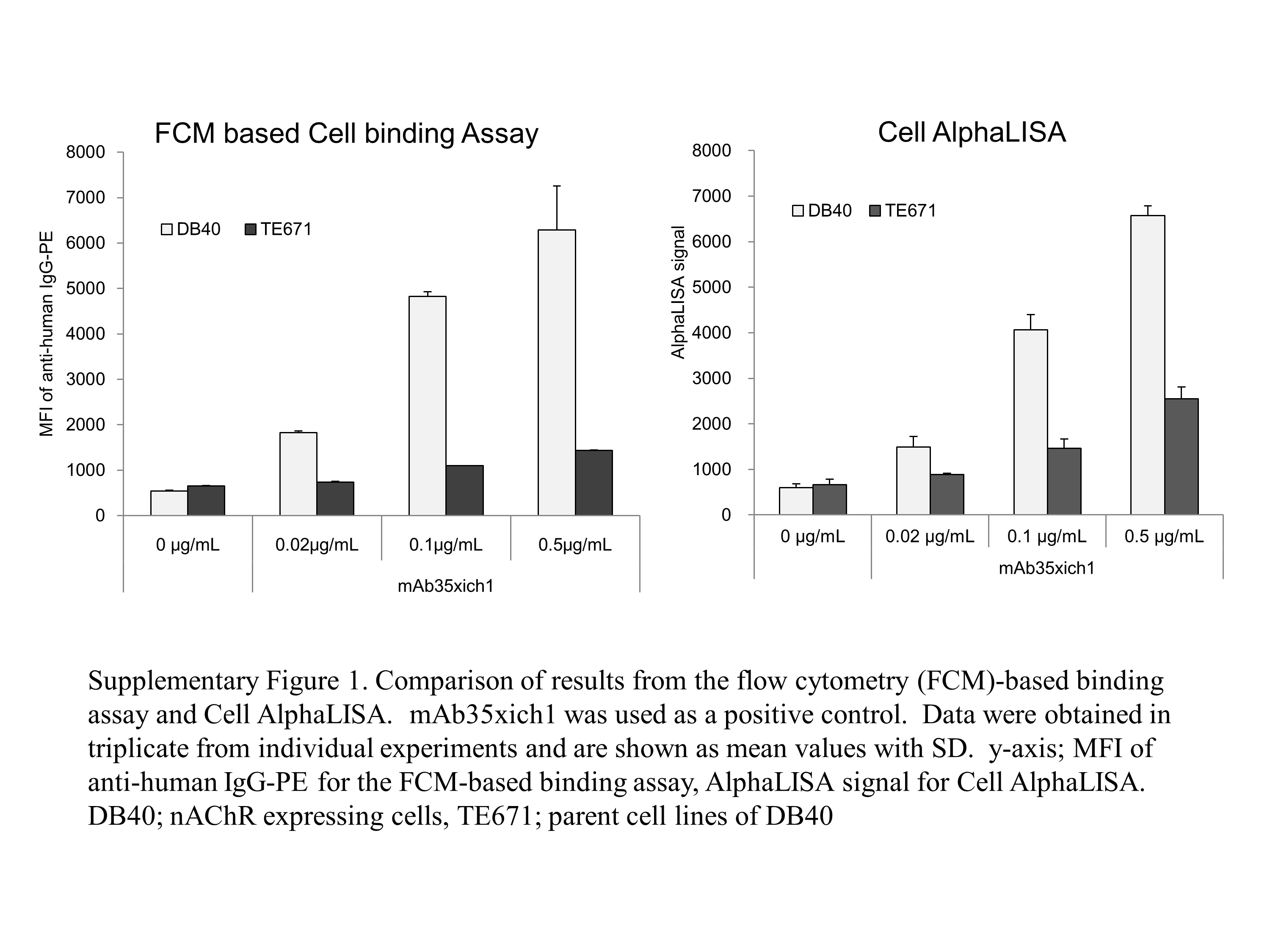

Supplement: S1 Fig — mAb35xich1 was used as a positive control. Data were obtained in triplicate from individual experiments and are shown as mean values with SD. y-axis; MFI of anti-human IgG-PE for the FCM-based binding assay, AlphaLISA signal for Cell AlphaLISA. DB40; nAChR expressing cells, TE671; parent cell lines of DB40 (TIF) [file pone.0185976.s001.TIF]

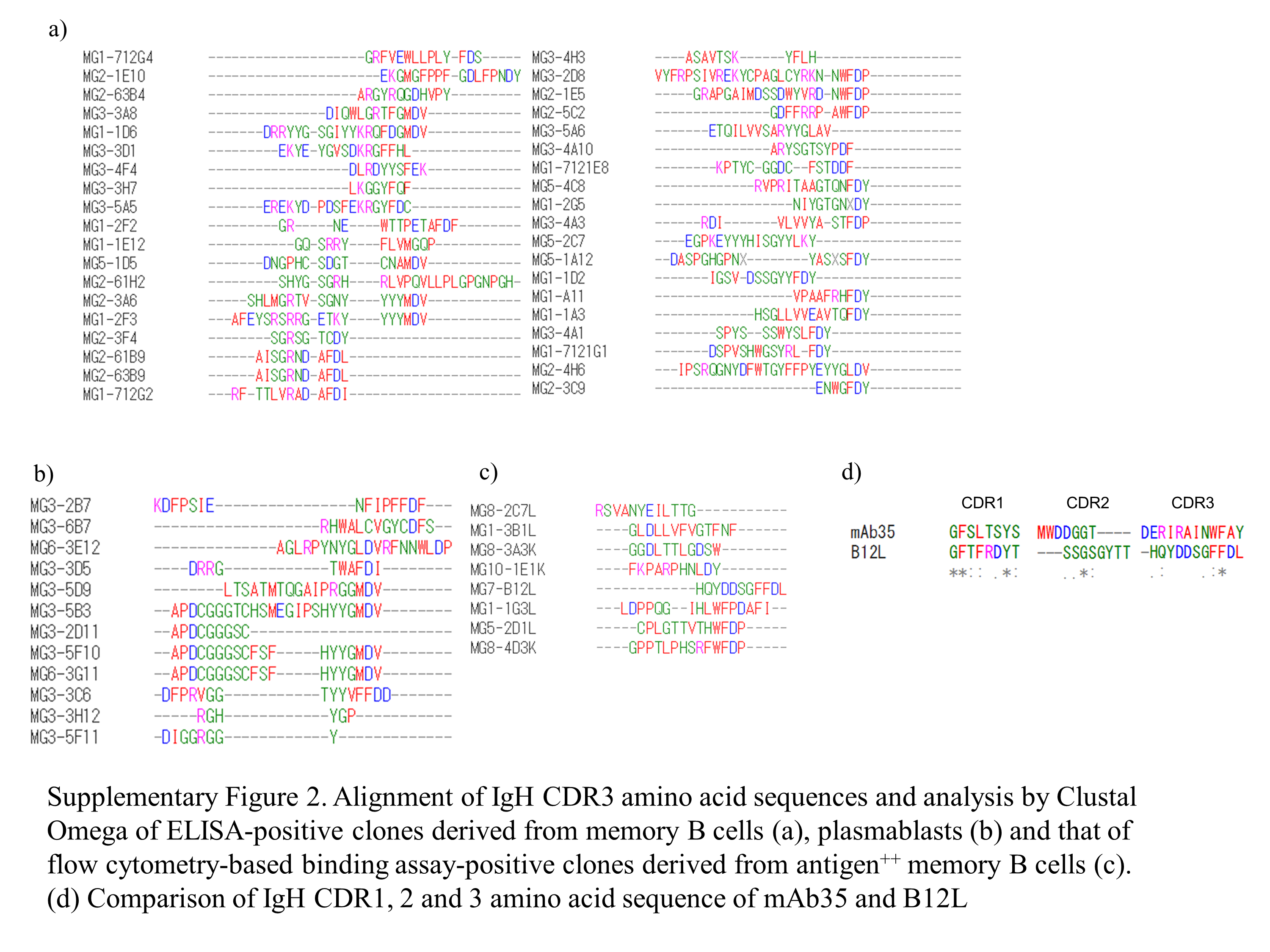

Supplement: S2 Fig — Clone IDs and amino acid sequences shown are derived from memory B cells (a), plasmablasts (b) and antigen++ memory B cells (c). (d) Comparison of amino acid sequences of CDR1, 2 and 3 in IgH between mAb35 and B12L are shown. (TIF) [file pone.0185976.s002.TIF]

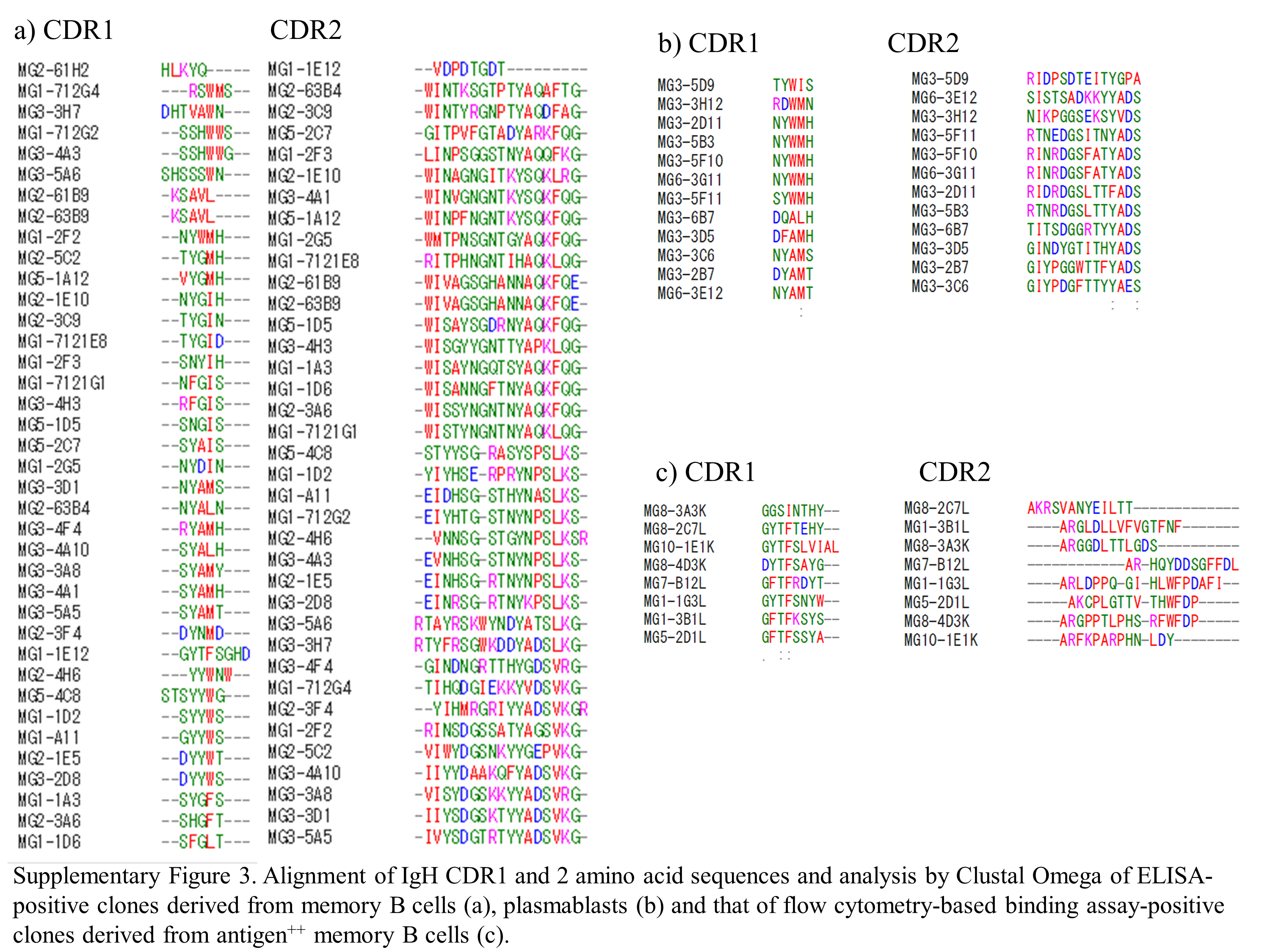

Supplement: S3 Fig — Alignment of IgH CDR1 and 2 amino acid sequences and analysis by Clustal Omega of ELISA-positive clones derived from memory B cells (a), plasmablasts (b) and that of flow cytometry-based binding assay-positive clones derived from antigen++ memory B cells (c). (TIF) [file pone.0185976.s003.TIF]
