# Supplementary material for: Analysis of peripheral B cells and autoantibodies against the anti-nicotinic acetylcholine receptor derived from patients with myasthenia gravis using single-cell manipulation tools
Source: PLoS One. 2017 Oct 17;12(10):e0185976. doi: 10.1371/journal.pone.0185976 (PMC5645109; doi:10.1371/journal.pone.0185976)
Supplement: S4 Fig — Alignment of IgL CDR1 and 2 amino acid sequences and analysis by Clustal Omega of ELISA-positive clones derived from memory B cells (a), plasmablasts (b) and that of flow cytometry-based binding assay-positive clones derived from antigen++ memory B cells (c). (PDF) [file pone.0185976.s004.pdf]

a)

CDR1		CDR2		CDR3	
MG1-A11	-------KLGDKF----	MG3-3A8	------------LQY	MG3-3D11	--QQYYLY------------
MG1-7121G4	----------ALPKQY-	MG2-5C2	---------EDR---	MG3-5A5	--QQYIYY------------
MG2-63B4	----------ILAKQY-	MG1-7211G2	------TNQTQRLR-	MG5-1A12	-RQEYSYS-RWT--------
MG1-7211G2	VASMLV-ATG-------	MG1-2F3	-----------DFF-	MG2-1E10	QWWDYHSD------------
MG1-2G5	--------SGINVGFYR	MG1-1D2	---GTY---------	MG3-3H7	QWWDYHSD------------
MG2-61H2	----------HNVGNY-	MG2-61B9	VGTGGIVG-------	MG1-1E12	-LQQAH--------------
MG1-1E12	--ERLDPRDGITS----	MG2-63B9	VGTGGIVG-------	MG3-4H3	--HQYGGS------------
MG1-1A3	-QYIVHGEDGNIY----	MG2-1E10	----------DDY--	MG5-2C7	--QQYKS-------------
MG1-2F2	-QGLVH-SDGNTY----	MG3-3H7	---------SDDY--	MG5-1D5	--QQYSSF------------
MG1-7211G1	-QSLVH-SDGNIY----	MG2-3A6	----------NDL--	MG2-1E5	--QQYHS-------------
MG2-4H6	-QSLLH-TNGYNY----	MG3-2D8	---------FDDL--	MG2-3C9	--QQYGYS-P----------
MG3-3A8	-----S-TMGDNY----	MG1-7211E8	--------DTR----	MG2-3F4	--QQYGS-------------
MG1-7211E8	-------STGAVTSGHS	MG1-A11	-------QDTK----	MG3-4A1	--QQYGSS-P----------
MG5-1A12	---------QSIREY--	MG2-63B4	-------KDT-----	MG2-4H6	-----MQALQTP--------
MG5-1D5	---------QSLSRW--	MG3-5A6	--------DTS----	MG1-2F2	-----MQATHRP--------
MG3-3D11	---------QTISSW--	MG2-61H2	--------DTS----	MG1-1A3	-----MQNTHWP--------
MG2-1E5	---------QSISSW--	MG2-4H6	-------LGS-----	MG3-5A6	-QQSYRAP------------
MG3-5A5	---------QSISSW--	MG3-4A3	-------EGS-----	MG3-4A3	--CSYAGRSTL---------
MG5-2C7	---------QSISSW--	MG1-2G5	-----YKSDSDK---	MG5-4C8	--CSYAGTYTW---------
MG3-4H3	--------SQSVSSNY-	MG1-7121G4	-------KDS-----	MG3-3A8	-----------EHGITA---
MG2-3C9	---------QSISSSY-	MG5-1A12	-------TDS-----	MG2-63B4	----YSAA---DN-------
MG2-3F4	---------QSVSSSY-	MG1-1E12	-------EVS-----	MG2-61B9	---------GTDHGTRSNFV
MG3-4A1	---------QSVSSSY-	MG3-4F4	-------EVS-----	MG2-63B9	---------GTDHGTRSNFV
MG3-5A6	----------QTTAGF-	MG1-2F2	-------MVS-----	MG2-5C2	--SSYVGS------------
MG2-1E10	----------QIGGRG-	MG5-4C8	-------DVS-----	MG3-4F4	--SSYTSSSTW---------
MG3-3H7	----------QIGGRG-	MG2-3C9	-------GAA-----	MG2-3A6	--------ATWDDNL-----
MG1-1D2	--------SSNIGAGYD	MG2-3F4	-------GAS-----	MG3-2D8	--------ATWDDRLN----
MG1-2F3	--------RSNIGAGYD	MG3-4A1	-------GAS-----	MG1-A11	--------QAWDSSTA----
MG3-2D8	--------SSNIGNNA-	MG3-4H3	-------AAS-----	MG1-7211G1	-----MPGSLWP--------
MG1-1D6	--------SSNIGRNT-	MG3-5A5	-------RAS-----	MG1-7211E8	-------LLSYNDA-R----
MG2-3A6	--------SLNIGRNY-	MG5-1D5	-------RAS-----	MG1-2G5	--------MIWENSA-----
MG5-4C8	---------SDVGGHNH	MG2-1E5	-------KAS-----	MG1-7211G2	--------MIWHSSA-----
MG3-4F4	--------SSDIGSYNR	MG3-3D11	-------KAS-----	MG1-1D6	--------AS----------
MG2-61B9	---------SDYSNYK-	MG5-2C7	-------KAS-----	MG2-61H2	---------SSVSTGRS---
MG2-63B9	---------SDYSNYK-	MG1-1D6	---------SNN---	MG1-7121G4	--------QSTDRSG-----
MG2-5C2	--------SSDVDV---	MG1-7211G1	-------KVS-----	MG1-1D2	--------QSHDSGL-----
MG3-4A3	--------SSDVRSYKF	MG1-1A3	-------KVSN----	MG1-2F3	--------QSYDSSLRG---

Supplementary Figure 4. Alignment of IgL CDR1 and 2 amino acid sequences and analysis by Clustal Omega of ELISA-positive clones derived from memory B cells (a), plasmablasts (b) and that of flow cytometry-based binding assay-positive clones derived from antigen^{++} memory B cells (c).

b) CDR1 / CDR2 / CDR3

CDR1 clone	CDR1	CDR2 clone	CDR2	CDR3 clone	CDR3
MG3-6B7	-------SGHSNHL	MG3-6B7	--GGNY	MG3-3D5	TSYTSSDNW---
MG3-3C6	QSLVHSDDGNTY--	MG3-5F11	YWGY--	MG6-3G11	-QYSG--TF---
MG3-3H12	QSVGGH--------	MG3-5D9	--GAS-	MG3-6B7	------ETWDSH
MG3-5F10	ESLQGSH-GYSY--	MG6-3E12	--AAS-	MG6-3E12	---QQANSFP--
MG3-2B7	QSLVHHN-GRTY--	MG6-3G11	--DAS-	MG3-2B7	---LQATQFP--
MG3-5F11	QSLLHSN-GNYF--	MG3-2D11	---LSS	MG3-3C6	---LQATQFP--
MG3-2D11	QSLLRSN-GHKY--	MG3-5F10	---LGS	MG3-5F11	---MQALQTP--
MG3-5B3	QSLLGSF-GHNY--	MG3-5B3	---VTS	MG3-2D11	---MQGLETP--
MG3-3D5	----SSDIGSYNR-	MG3-3D5	--EVT-	MG3-5B3	---MQGLEPP--
MG6-3E12	-----QGIRTW---	MG3-2B7	--EVS-	MG3-5F10	---MQGLQSP--
MG3-5D9	-----QSIRSTY--	MG3-3C6	--KVS-	MG3-3H12	---QQYHDWP--
MG6-3G11	-----QGISS----	MG3-3H12	--DVS-	MG3-5D9	---QQFDDS---

c) CDR1 / CDR2 / CDR3

CDR1 clone	CDR1	CDR2 clone	CDR2	CDR3 clone	CDR3
MG8-3A3K	-------SQTVSSY---	MG10-1E1K	-KVS	MG8-4D3K	STSVPRTL--------
MG10-1E1K	QALVHSDANIY------	MG1-3B1L	YDDT	MG10-1E1K	---MPGSWP-------
MG8-4D3K	-------ENISGSY---	MG1-1G3L	-GNT	MG1-3B1L	---------CSYVGRS
MG5-2D1L	------SSNIGAGYD--	MG5-2D1L	-GNT	MG8-3A3K	-----QRYG-------
MG8-2C7L	------SSNIGAGYD--	MG8-2C7L	-GNN	MG1-1G3L	-----QSYDNSLSG--
MG7-B12L	------SANIGSHF---	MG7-B12L	RGS-	MG5-2D1L	-----QSYDNSLSG--
MG1-1G3L	-----------GTAPKL	MG8-3A3K	-GAS	MG8-2C7L	-----QAYDRSLSG--
MG1-3B1L	------NSDI-GTYKL-	MG8-4D3K	-GAS	MG7-B12L	-----ATWDDSLS---

Supplementary Figure 4 (continued). Alignment of IgL CDR1 and 2 amino acid sequences and analysis by Clustal Omega of ELISA-positive clones derived from memory B cells (a), plasmablasts (b) and that of flow cytometry-based binding assay-positive clones derived from antigen^{++} memory B cells (c).
