# Supplementary material for: Analysis of peripheral B cells and autoantibodies against the anti-nicotinic acetylcholine receptor derived from patients with myasthenia gravis using single-cell manipulation tools
Source: PLoS One. 2017 Oct 17;12(10):e0185976. doi: 10.1371/journal.pone.0185976 (PMC5645109; doi:10.1371/journal.pone.0185976)
Supplement: S1 Table — (DOCX) [file pone.0185976.s005.docx]

**S1 Table. Age, sex, serological data, clinical symptoms and MGFA classification of MG donors enrolled in this study**

Donor ID Age Gender anti-AChR antibody titer Clinical Symptoms MGFA classification

(nmol/L) The current condition /

The condition at its worst

　　　 Informatin of treatment history

MG1 55 Female 160 Severe/Severe IVa

Immune treatment: 10 mg/day of prednisolone, 3mg/day of tacrolimus, and 5-6 times/year of plasmapheresis combined with intravenous methylprednisolone

MG2 44 Female 80-90 Severe/Severe IVa

Immune treatment: 5-10 mg/day of prednisolone, 3mg/day of tacrolimus, and 5-6 times/year of plasmapheresis combined with intravenous methylprednisolone

MG3 48 Female 53 Moderate/Moderate IIIa

Immune treatment: 10 mg/day of prednisolone, 3mg/day of tacrolimus.

MG5 26 Female 650 Non/Moderate IIb

Immune treatment: 3mg/day of tacrolimus.

MG6 28 Female 28 Non/Moderate IIb

Immune treatment: 1.5mg/day of tacrolimus

MG7 34 Male 27 Moderate/Severe IVb

Immune treatment: 5 mg/day of prednisolone, 3mg/day of tacrolimus

MG8 72 Female 2.3 Moderate/Moderate IIIa

Immune treatment: 5 mg/day of prednisolone, 3mg/day of tacrolimus, and 2-3 times/year of plasmapheresis combined with intravenous methylprednisolone

MG10 46 Female 65 Severe/Severe V

Immune treatment: 10 mg/day of prednisolone, 3mg/day of tacrolimus, and 8-9 times/year of plasmapheresis combined with intravenous methylprednisolone

MG11 70 Female 7.1 Non/Moderate IIIa

Immune treatment: 5 mg/day of prednisolone, 3mg/day of tacrolimus
