# Supplementary material for: Analysis of peripheral B cells and autoantibodies against the anti-nicotinic acetylcholine receptor derived from patients with myasthenia gravis using single-cell manipulation tools
Source: PLoS One. 2017 Oct 17;12(10):e0185976. doi: 10.1371/journal.pone.0185976 (PMC5645109; doi:10.1371/journal.pone.0185976)
Supplement: S2 Table — Number and percentage of IgG genes amplified from a) peripheral memory B cells derived from MG donors, b) peripheral plasmablasts derived from MG donors, c) peripheral antigen++ memory B cells derived from MG donors. (DOCX) [file pone.0185976.s006.docx]

**S2 Table. Number and percentage of genes amplified from^[[1]](#footnote-1)^**

1. **peripheral memory B cells derived from MG donors and healthy control (HC)**

ID MG1 MG2 MG3 MG5 MG7 HC

gene No of % of No of % of No of % of No of % of No of % of No of % of genes amplified

γ 208 59 370 42 467 82 340 60 282 59 242 64

κ 160 46 379 43 254 45 306 54 160 33 150 40

λ 169 48 383 43 327 57 225 39 267 56 230 60

- 50 14 101 11 24 4 55 10 53 11 20 5

IgH/IgL 208 59 414 47 497 87 346 61 269 56 250 66

N 351 100 883 100 570 100 570 100 480 100 380 100

**b) peripheral plasmablasts derived from MG donors**

ID MG1 MG3 MG6

gene No of % of No of % of No of % of genes amplified

γ 20 21 246 46 128 31

κ 66 69 291 54 166 40

λ 63 66 353 65 281 68

- 20 21 49 9 44 11

IgH/IgL 26 27 326 60 152 37

N 96 100 540 100 414 100

**c) peripheral antigen^++^ memory B cells derived from MG donors**

ID MG1 MG5 MG7 MG8 MG10 MG11

gene No of % of No of % of No of % of No of % of No of % of No of % of genes amplified

γ 173 60 180 69 148 45 185 64 185 55 34 33

κ 102 35 113 44 187 57 94 32 96 29 33 32

λ 193 67 176 68 136 41 180 62 221 66 41 39

- 24 8 21 8 49 15 26 9 38 11 25 24

IgH/IgL 198 69 219 85 178 54 188 65 192 57 40 38

N 288 100 259 100 329 100 291 100 336 100 104 100

1. ID indicates the donor enrolled in this study, HC indicates healthy control, γ; gamma heavy chain gene, κ; kappa light chain gene, λ; lamda light chain gene, -; no amplification, IgH/IgL indicates the clones successfully amplified with paired heavy and light chains, N; total number of clones [↑](#footnote-ref-1)
